# Chronic maternal exposure to titanium dioxide nanoparticles alters breathing in newborn offspring

**DOI:** 10.1186/s12989-022-00497-4

**Published:** 2022-08-18

**Authors:** Eloïse Colnot, Laura Cardoit, Marie-Jeanne Cabirol, Lydia Roudier, Marie-Helene Delville, Anne Fayoux, Muriel Thoby-Brisson, Laurent Juvin, Didier Morin

**Affiliations:** 1 Univ. Bordeaux, CNRS, INCIA, UMR 5287, F-33000 Bordeaux, France; 2 Univ. Bordeaux, CNRS, Bordeaux INP, ICMCB, UMR 5026, F-33608 Pessac, France; 3 Univ. Bordeaux, Department of Health, Safety and Environment, Bordeaux Institute of Technology, F-33175 Gradignan, France

**Keywords:** Titanium dioxide nanoparticles, Maternal exposure, Respiration, Mouse, Nervous system development, Neurotoxicity

## Abstract

**Background:**

Over the last two decades, nanotechnologies and the use of nanoparticles represent one of the greatest technological advances in many fields of human activity. Particles of titanium dioxide (TiO_2_) are one of the nanomaterials most frequently found in everyday consumer products. But, due in particular to their extremely small size, TiO_2_ nanoparticles (NPs) are prone to cross biological barriers and potentially lead to adverse health effects. The presence of TiO_2_ NPs found in human placentae and in the infant meconium has indicated unequivocally the capacity for a materno-fetal transfer of this nanomaterial. Although chronic exposure to TiO_2_ NPs during pregnancy is known to induce offspring cognitive deficits associated with neurotoxicity, the impact of a gestational exposure on a vital motor function such as respiration, whose functional emergence occurs during fetal development, remains unknown.

**Results:**

Using in vivo whole-body plethysmographic recordings from neonatal mice, we show that a chronic exposure to TiO_2_ NPs during pregnancy alters the respiratory activity of offspring, characterized by an abnormally elevated rate of breathing. Correspondingly, using ex vivo electrophysiological recordings performed on isolated brainstem-spinal cord preparations of newborn mice and medullary slice preparations containing specific nuclei controlling breathing frequency, we show that the spontaneously generated respiratory-related rhythm is significantly and abnormally accelerated in animals prenatally exposed to TiO_2_ NPs. Moreover, such a chronic prenatal exposure was found to impair the capacity of respiratory neural circuitry to effectively adjust breathing rates in response to excitatory environmental stimuli such as an increase in ambient temperature.

**Conclusions:**

Our findings thus demonstrate that a maternal exposure to TiO_2_ NPs during pregnancy affects the normal development and operation of the respiratory centers in progeny.

**Supplementary Information:**

The online version contains supplementary material available at 10.1186/s12989-022-00497-4.

## Background

During the last decade, a new concept, termed ‘exposome’ has emerged in epidemiology in order to improve environmental exposure assessment [1]. The exposome is composed of all exposures to which an individual is subjected, from conception to death, including both internal (such as metabolism, body morphology, or physical activity) and external factors (such as climate, radiations, infectious agents, or chemical contaminants and environmental pollutants) including nanoparticles (NPs). Indeed, since the beginning of this century, the use of NPs has undergone a rapid growth until nowadays. With the development and expansion of NPs into different fields of human activity (electronics, energy, clothes, food processing industry, cosmetics, medicine, etc.), nanosized materials are permanently present in our environment. Among synthetic nanomaterials, titanium dioxide (TiO_2_) is one of the most commonly used and is found in everyday consumer products [2–4]. Given their ubiquitous employment at widely variable doses, TiO_2_ NPs have been recently found to have the greatest potential impact on human health [5]. Questions then arise concerning the impact of their possible accumulation in biological tissues and their potential toxicity, especially when exposure occurs during vulnerable periods of life such as pregnancy.

Regardless of the maternal route of exposure during gestation (i.e. ingestion, inhalation, injection), TiO_2_ NPs can induce adverse health effects in progeny. Recently, it has been clearly established that exposure to TiO_2_ NPs through ingestion during pregnancy impairs placentation (in mice [6]) and increases titanium concentrations in placental tissue (in rats [7]). There is strong evidence, moreover, that such a prenatal ingestion exposure to TiO_2_ NPs interferes with the normal development of offspring. For example in rodents, maternal TiO_2_ exposure can inhibit the development of the fetal skeleton by reducing ossification, and can lead to an increase in the number of fetuses with dysplasia [8]. Such a mode of exposure can also alter lung and brain development, increasing the number of apoptotic lung cells [9] and causing impaired learning and memory capability [10], respectively. By inhalation during pregnancy, maternal exposure to TiO_2_ NPs can also impair fetal heart and lung development, which is characterized by cardiac contractile dysfunction (in rats [11] and mice [12]) and a reduction in the number of offspring lung alveoli (in mice [13]). Finally, following subcutaneous injection, the transfer of TiO_2_ NPs from the mother during pregnancy can alter different components of the offspring’s nervous system (in mice [14, 15]), leading to neurobehavioral diseases in the prenatally-exposed adult (in rats [16]). However, there remains a lack of information regarding the effects in progeny of a chronic prenatal exposure to TiO_2_ NPs on vital motor functions, such as breathing, and on the development and operation of the neural networks engaged in respiratory rhythmogenesis. In mice, for example, the central neural rhythm underlying respiration, which originates from a medullary region called the pre-Bötzinger complex, emerges during the last third of gestation (for a review see references [17, 18]). Thus, chemical exposure-induced abnormalities in the development of respiratory neural control are likely to have a major impact, and may contribute to neonatal morbidity and mortality.

By combining chemical analysis, physiological and neurophysiological in vivo and ex vivo experiments in newborn mice, we aimed at studying the impact of a chronic prenatal exposure to TiO_2_ NPs on the early post-natal functioning of the neural networks (or respiratory centers) responsible for breathing. We report deleterious influences of TiO_2_ NPs on respiration in newborn offspring, leading to an abnormally elevated respiratory rhythm frequency, which in turn impairs the ability of the central respiratory neural network to satisfactorily respond to excitatory environmental stimuli.

## Results

### Physicochemical characterization of titanium dioxide nanoparticles

**Fig. 1:**
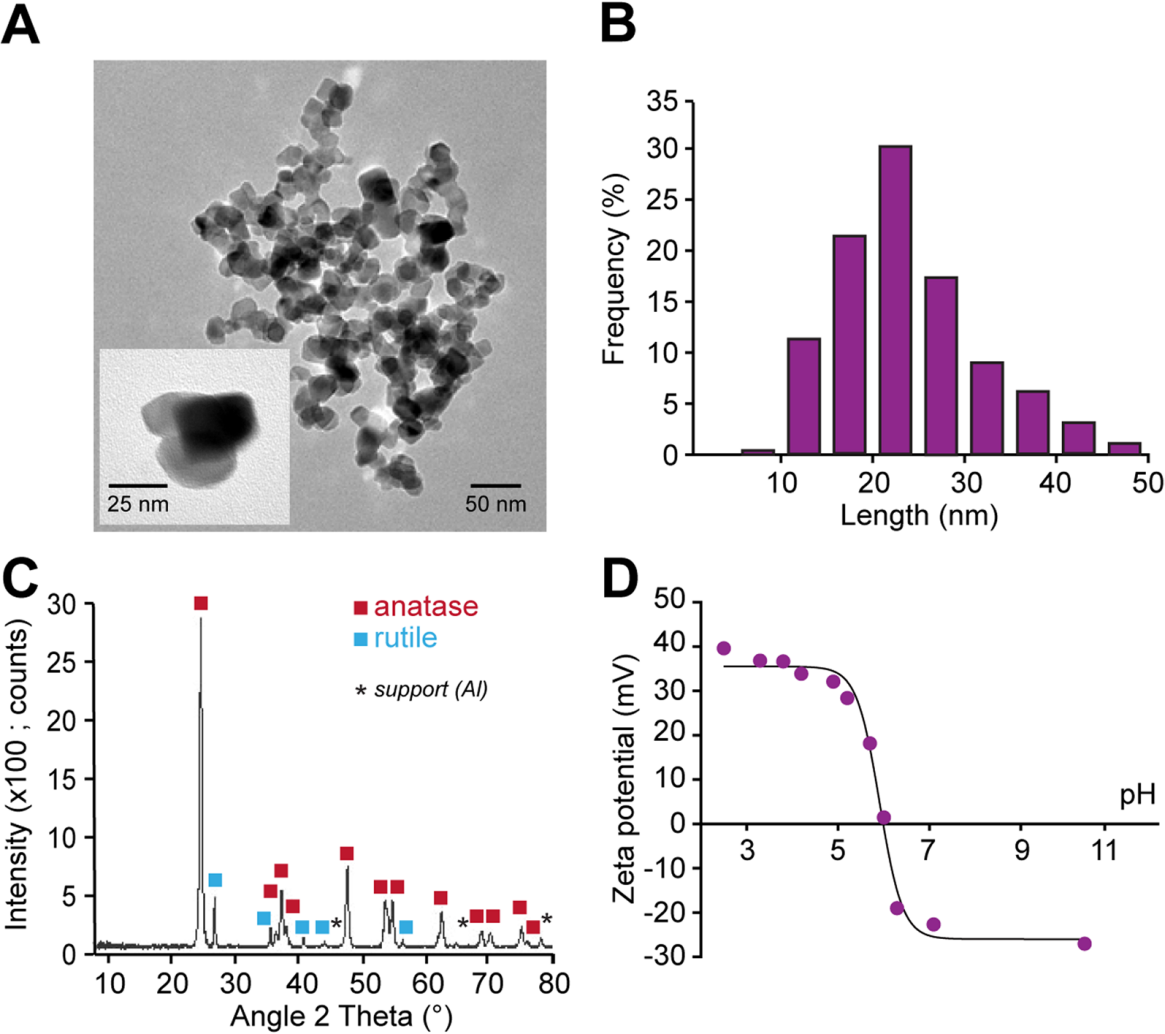
Physicochemical characterization of TiO_2_ NPs. **A** Transmission electron microscopy image of TiO_2_ NPs. **B** Bar chart showing TiO_2_ NP size distribution based on 255 NPs. **C** X-ray diffraction pattern of TiO_2_ NPs. **D** Scatter plot showing the relationship between Zeta potential and pH. Al, aluminum

The batch of TiO_2_ NPs used in this study was fully characterized as follows. In an initial step, transmission electron microscopy (TEM) was employed to confirm the size and polydispersity of the nanomaterials (Fig. 1A) and that individual particle size measured on more than 250 NPs ranged from 5 to 50 nm (Fig. 1B), with an average value of 24 ± 8 nm. In a second step, X-ray diffraction pattern, which enabled distinguishing the different crystalline forms of TiO_2_ NPs, indicated an anatase/rutile composition with a high proportion of anatase phase (80/20, respectively; Fig. 1C). In a third step, we quantified the surface charge of particles used in our experiments. The zeta potential of TiO_2_ NPs was measured as a function of pH, and a mean value of − 25 mV was detected (Fig. 1D) at a physiological neutral pH (= 7.4).

### Effects of TiO_2_ NPs on breathing in prenatally-exposed neonates

**Fig. 2:**
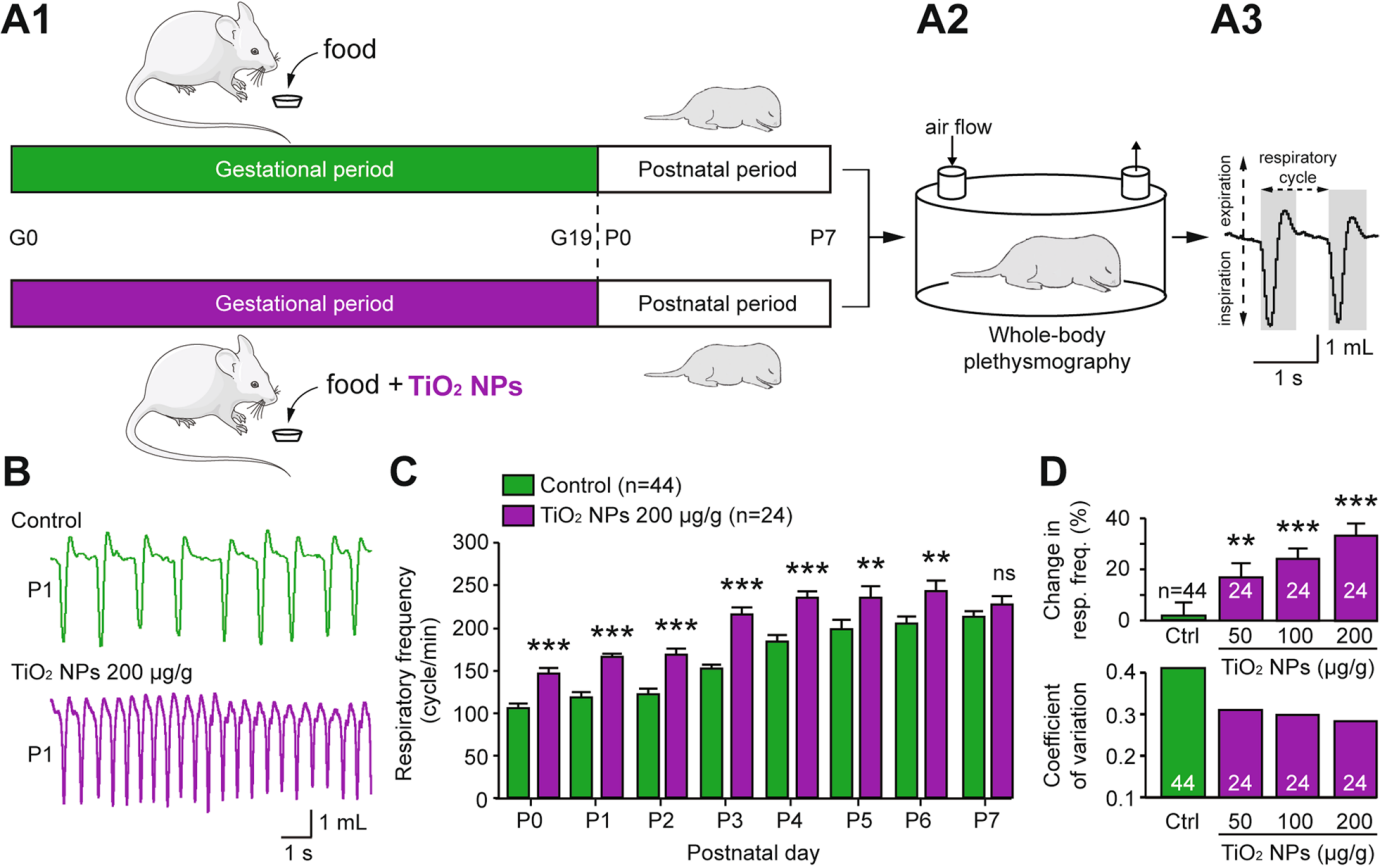
Breathing frequency is abnormally elevated in neonates that have been prenatally exposed to TiO_2_ NPs. **A1** Schematics of protocols for non-exposed (green) and TiO_2_ NP-exposed (purple) pregnant mice during the gestational (G) period, and **A2** subsequent whole-body plethysmography experiments performed on offspring during the first postnatal (P) week. **A3** Expanded trace of the plethysmograph box pressure signal derived from a control animal. **B** Whole-body plethysmographic recordings of non-exposed (green), TiO_2_ NP-exposed (purple) neonates obtained at P1. **C** Bar charts showing the variation in spontaneous respiratory frequency (mean ± SEM) in non-exposed (green bars) and prenatally TiO_2_ NP-exposed (200 µg/g; purple bars) animals during the first postnatal week. **D** Bar charts illustrating the dose-dependent effect of gestational exposure to TiO_2_ NPs on respiratory frequency (top) and mean coefficients of variation under the four experimental conditions (bottom). Data were pooled from P0 to P6. The number of animals is indicated in each bar. **p < 0.01; ***p < 0.001. The mouse image is from Servier Medical Art website (smart.servier.com)

**Fig. 3:**
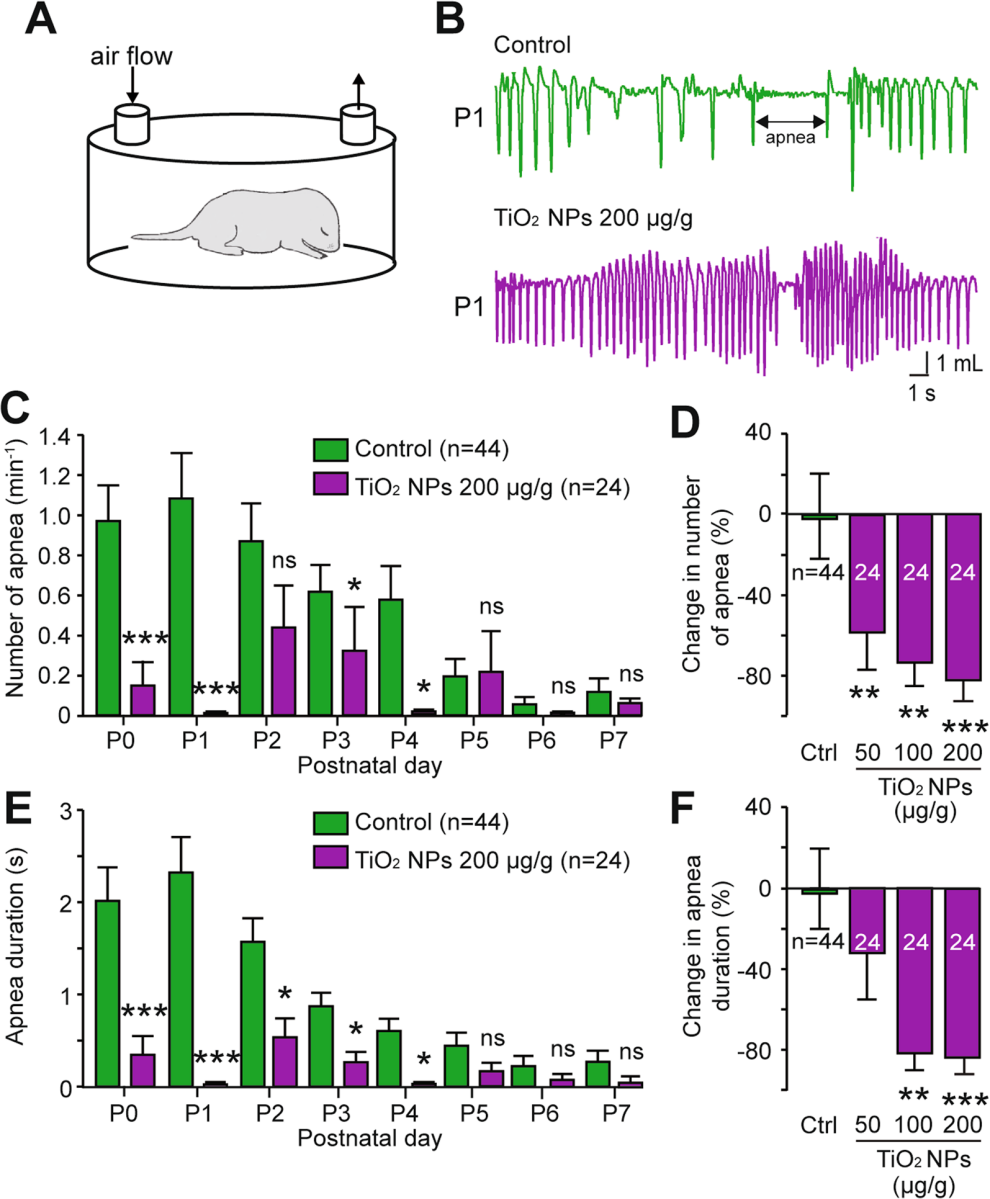
The number and duration of apneas are significantly lower in prenatally-exposed offspring. **A** Schematic of experimental procedure. **B** Whole-body plethysmographic recordings of non-exposed (top green) and TiO_2_ NP-exposed (bottom purple) neonates obtained at P1. **C**, **E** Bar charts showing the variation in the number (**C**) and duration (**E**) of apneas in non-exposed (green bars) and prenatally TiO_2_ NP (200 µg/g)-exposed (purple bars) groups during the first postnatal week (mean ± SEM). **D**, **F** Bar charts illustrating the dose-dependent effect of a gestational exposure to TiO_2_ NPs on the number (**D**) and duration (**F**) of apneas under the four experimental conditions. Data were pooled from P0 to P6. The number of animals is indicated in each bar. *p < 0.05; **p < 0.01; ***p < 0.001

Throughout the entire gestational period, which lasts 19 days in the mouse line used in the present study, 3 groups of pregnant mice were exposed daily to food containing different TiO_2_ NPs concentrations (50, 100 and 200 µg/g body weight), while a further group of unexposed animals served as control (Fig. 2A1). Importantly, the repeated exposure to TiO_2_ NPs during gestation affected neither the weight gain trajectory of pregnant mice nor the number of neonates produced per litter (Additional file 1: Fig. S1A–C). To assess the effects of a chronic prenatal exposure to TiO_2_ NPs on offspring breathing, whole-body plethysmographic recordings were performed during the first postnatal week (Fig. 2A2). This non-invasive quantitative technique allowed monitoring in awake unrestrained pups both inspiratory and expiratory phases of respiration, which appear as downward and upward deflections, respectively, on the recording trace (Fig. 2A3–B). As reported previously [19], the breathing rate of non-preexposed neonatal animals increased significantly over the first postnatal week, ranging from 106 ± 6 cycles/min at postnatal day 0 (P0) to 212 ± 8 cycles/min at P7 (n = 44 animals; p < 0.001; Fig. 2C). Although a similar breathing rate increase was observed in the prenatally-TiO_2_ NPs exposed (200 µg/g) group (from 146 ± 6 cycles/min at P0 to 228 ± 9 cycles/min at P7; n = 24 animals; p < 0.001; Fig. 2C), the respiratory frequency was significantly higher on each day compared to non-exposed animals (Fig. 2B) during the 6 first postnatal days (Fig. 2C). Importantly, these effects on breathing cannot be attributable to a change in pup morphology or to a structural alteration of their lung tissue, as there were no significant differences in the weight and size of the prenatally exposed offspring compared to control (Additional file 1: Fig. S1D, E), and no significant differences in the lung morphology in both groups (Additional file 2: Fig. S2). Furthermore, the abnormal excitatory effect of prenatal TiO_2_ NP exposure on postnatal breathing rate occurred in a dose-dependent manner (Fig. 2D, top). For example, in the TiO_2_ NPs (200 µg/g)-exposed group (n = 24 animals), the respiratory frequency was 33 ± 5% higher than in control neonates (n = 44; p < 0.001). Moreover, the breath-to-breath variability within a given animal, expressed as a coefficient of variation, decreased from 0.40 to 0.28 in non-exposed and TiO_2_ NPs (200 µg/g)-exposed animals, respectively (Fig. 2D, bottom). In this context, a further important comparative observation was that in non-exposed animals, episodes of pauses in breathing, or apneas (Fig. 3B, green), occurred at P0 with a recurrence of 0.97 ± 0.2 per minute (n = 44 animals; Fig. 3C), and with a mean duration of 2.0 ± 0.4 s (Fig. 3E). After this initial frequent expression in control neonates, however, apneas had almost disappeared by the end of the first postnatal week (at P7: 0.13 ± 0.3 apneas/min (Fig. 3C) with a mean duration of 0.27 ± 0.1 s (Fig. 3E)). Strikingly as illustrated in the sample plethysmographic recording of Fig. 3B (purple), this characteristic of normal postnatal breathing development, which directly affects the degree of breath-to-breath interval variability (see Fig. 2D, bottom), was nearly absent at birth in prenatally TiO_2_ NPs (200 µg/g)-exposed offspring (at P0: 0.15 ± 0.1 apneas/min; n = 24 animals (Fig. 3C); mean duration 0.35 ± 0.2 s (Fig. 3E)). Here again, this effect of chronic gestational TiO_2_ NP exposure on the number and duration of apneas occurred in a clear dose-dependent manner (Fig. 3D, F, respectively). For example, the number and duration of apneas were fivefold lower in prenatally TiO_2_ NPs (200 µg/g)-exposed offspring (n = 24 animals) compared to non-exposed control (n = 44; p < 0.001). Taken together, these finding thus provide compelling evidences that a chronic prenatal exposure to TiO_2_ NPs impairs respiratory function in newborn mice.

### Maternal exposure to TiO_2_ NPs affects the neural control of offspring breathing

**Fig. 4:**
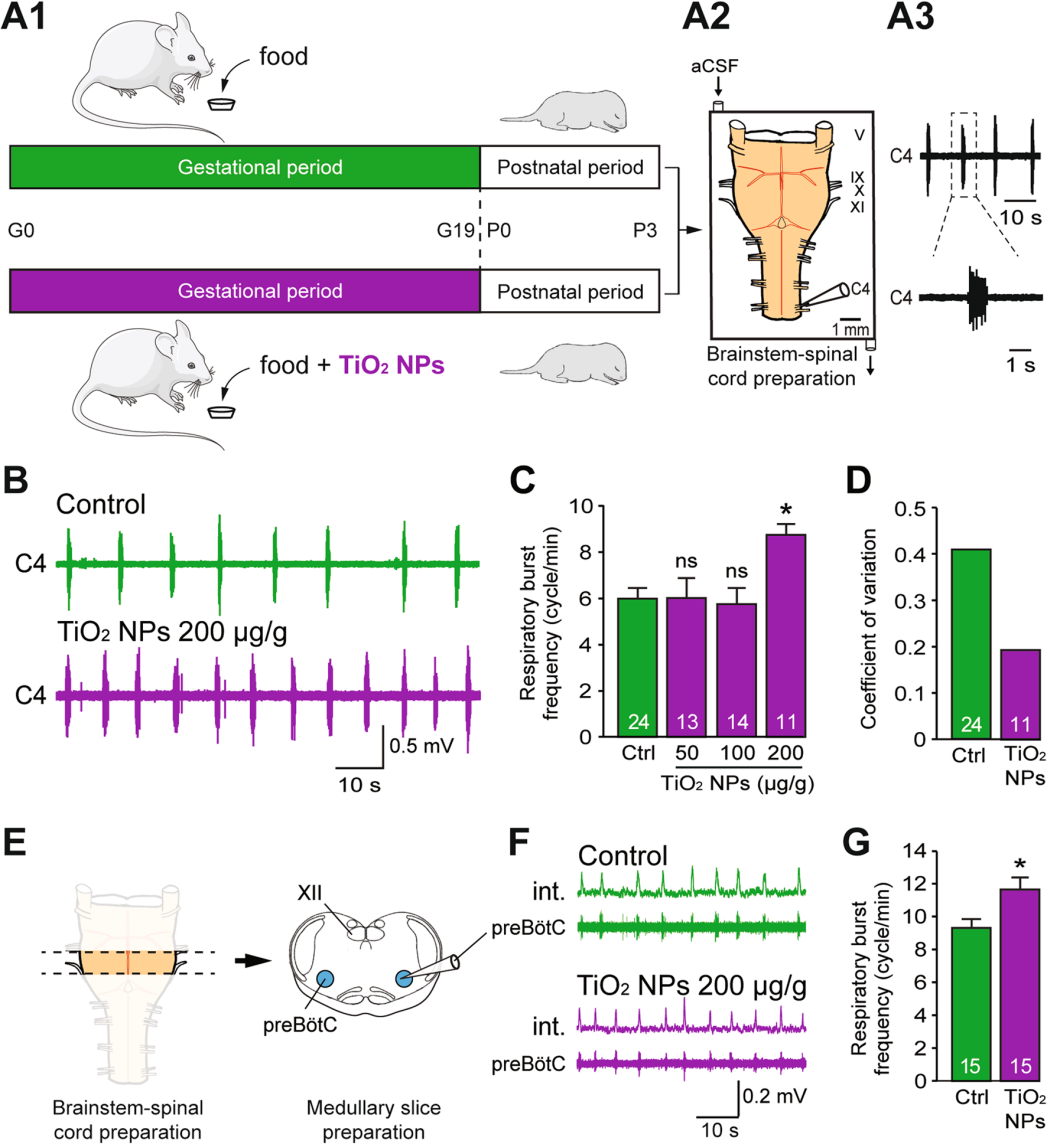
Effects of chronic maternal exposure to TiO_2_ NPs during gestation on respiratory-related motor burst activity in offspring ex vivo. **A1** Schematics of protocols for non-exposed (green) and TiO_2_ NP-exposed (purple) pregnant mice during the gestational (G) period, and subsequent electrophysiological experiments performed on isolated brainstem-spinal cord preparations (**A2**) from offspring during the three first postnatal (P) days. **A3** Raw spontaneous inspiratory-related burst activity recorded from a cervical (C4) spinal ventral root. **B** Raw extracellular recordings of spontaneous burst activity in a C4 ventral root of isolated preparations from unexposed (green) and prenatally TiO_2_ NP (200 µg/g)-exposed (purple) neonates obtained at P0. **C** Bar charts showing the variation in spontaneous burst frequency (mean ± SEM) in control (Ctrl, green bar) and prenatally TiO2 NP-exposed (purple bars) groups. Data were pooled from P0 to P3. The number of animals is indicated in each bar. **D** Mean coefficients of variation in cycle durations for the control and TiO_2_ NP (200 µg/g)-exposed groups. **E** Schematics of experimental procedures used to obtain a medullary slice preparation. **F** Integrated (int.) and raw extracellular burst activity recorded directly from the pre-Bötzinger complex (preBötC) in such slice preparations from unexposed (green) and prenatally TiO_2_ NP (200 µg/g)-exposed (purple) neonates. **G** Bar charts showing difference in spontaneous respiratory burst frequency (mean ± SEM) between control (Ctrl, green bar) and prenatally TiO_2_ NP-exposed groups (purple bar). *p < 0.05; aCSF, artificial cerebrospinal fluid; V, trigeminal nerves; IX, glossopharyngeal nerves; X, vagal nerves; XI, accessory nerves; XII, hypoglossal nuclei. The mouse image is from Servier Medical Art website (smart.servier.com)

The abnormally high respiratory rate observed in gestationally-exposed progeny following a materno-fetal transfer of nanoparticles could result from an action of TiO_2_ NPs on the central nervous system of pups. We hypothesised, therefore, that the particle-induced change in respiratory frequency of neonates could be the consequence of an abnormal excitatory state of the central pattern generator (CPG) neural circuitry for respiration. To test this possibility, we directly assessed the electrophysiological function of the respiratory CPG using isolated ex vivo brainstem-spinal cord preparations from neonatal animals (from P0 to P3; Fig. 4A1, 2), a reduced preparation that contains the neuronal circuits responsible for respiratory rhythmogenesis. In such ex vivo preparations, respiratory-related motor activity is expressed spontaneously, consisting of episodes of rhythmic motor burst discharge that can be recorded from spinal cervical C4 ventral roots (Fig. 4A3) that carry phrenic motor axons to the diaphragm (the main inspiratory muscle; see also [20]). Due to our particular experimental conditions (relatively low temperature, absence of sensory feedback, presence of pontine structures exerting an inhibitory influence on respiratory rhythm production) and as previously described [21–24], although the mean respiratory burst frequency was generally lower than that found in vivo, each C4 ventral root motor burst corresponded to the inspiratory phase of respiration [25]. In accordance with our observations from in vivo experiments, the frequency of spontaneous respiratory-related bursting activity recorded from prenatally TiO_2_ NP (200 µg/g)-exposed animals was significantly higher than in the non-exposed control group (8.7 ± 1.6 cycles/min (n = 11) *vs* 6.0 ± 2.4 cycles/min (n = 24), respectively; p < 0.05; Fig. 4B, C). Furthermore, we observed that the variability in burst-to-burst interval (expressed as the coefficient of variation) was significantly lower between treated and control preparations, decreasing from 0.40 to 0.19 in non-exposed and TiO_2_ NP (200 µg/g)-exposed animals, respectively (Fig. 4D). Already observed in in vivo experiments (Fig. 2D, bottom), a consequence of this regularization could be a loss in capacity of the respiratory system to produce adapted and efficient responses to changing internal or external stimuli (see below).

To directly test whether TiO_2_ NPs exposure exerts an influence on the respiratory central command, we used a transverse brainstem slice preparation (Fig. 4E) that isolates the pre-Bötzinger complex, a bilaterally-distributed network that is known to play a major role in generating the respiratory rhythm [26, 27]. Specifically, this structure, constitutes a neuronal circuit kernel that controls rhythmic lung ventilation in mammals [27, 28], and expresses spontaneous rhythmic bursts of circuit activity in slice preparations (Fig. 4F) that corresponds to the motor patterns of fictive inspiration recorded in brainstem-spinal cord preparations (see Fig. 4A3). In such slice preparations from prenatally TiO_2_ NP (200 µg/g)-exposed animals, the frequency of spontaneous inspiratory-related bursts was again significantly higher than in the non-exposed control group (11.6 ± 0.65 cycles/min (n = 15) vs 9.3 ± 0.6 cycles/min (n = 15), respectively; p = 0.014; Fig. 4F–G). Taken together, therefore, these findings from two levels of ex vivo isolation strongly support the conclusion that in mice, a chronic maternal exposure to TiO_2_ NPs can alter the prenatal development of breathing, at least in part by changing the excitability of the central neural networks that are actually responsible for rhythmogenesis in this critical rhythmic motor function.

### Drastically reduced respiratory responses to excitatory stimuli in prenatally TiO_2_ NP-exposed neonates

**Fig. 5:**
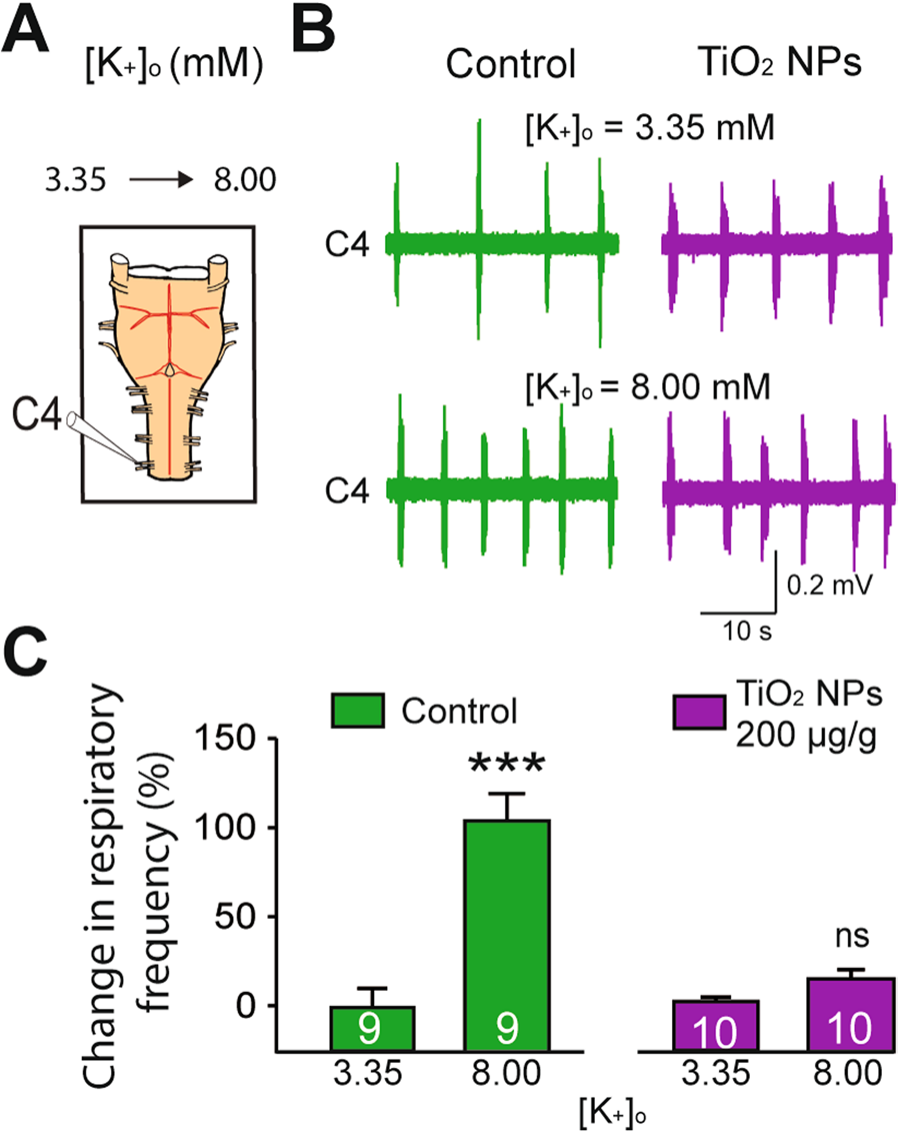
Lack of elevated potassium-induced excitation of the respiratory central pattern generator (CPG) in preparations from prenatally TiO_2_ NP-exposed offspring. **A** Schematic of experimental procedure. **B** Raw extracellular burst activity recorded from a C4 ventral root in isolated preparations from unexposed (Control, green traces) and prenatally TiO_2_ NP (200 µg/g)-exposed (purple traces) neonates during bath perfusion of saline with normal (3.35 mM, upper traces) and elevated (8.00 mM, lower traces) concentrations of K^+^. **C** Bar charts showing changes in spontaneous burst frequency (mean ± SEM) in control (green bars) and prenatally TiO_2_ NP-exposed (purple bars) groups under normal (3.35 mM) and elevated (8.00 mM) K^+^ saline. Data were pooled from P0 to P3. The number of animals is indicated in each bar. ***p < 0.001; ns, not statistically significant

The observation reported above that in both in vivo and ex vivo conditions, the variability in respiratory rate was clearly reduced in gestationally TiO_2_ NP-exposed offspring suggested that the flexibility and responsiveness of respiratory function to environmental constraints could be diminished in these animals. To assess this possibility, a series of experiments was performed using isolated brainstem-spinal cord preparations to examine the ability of respiratory CPG neural circuitry to produce adaptive responses to extrinsic excitatory stimulation. Because extracellular potassium (K^+^) concentration is critical in determining the resting membrane potential of neurons, a modified saline with an elevated K^+^ concentration (8.00 mM instead of 3.35 mM) was used as a depolarizing stimulus to thereby increase the excitability of the respiratory CPG networks (Fig. 5A). In non-exposed (control) brainstem-spinal cord preparations, the superfusion of saline with elevated K^+^ induced a significant 100% increase in respiratory-related burst frequency (from 5.4 ± 0.6 cycles/min (K^+^ 3.35 mM) to 10.8 ± 0.8 cycles/min (K^+^ 8.00 mM); n = 9; p < 0.001; Fig. 5B, C). On the other hand, the TiO_2_ NP (200 µg/g)-exposed group showed only a weak frequency augmentation which did not reach statistical significance (from 8.7 ± 0.6 cycles/min (K^+^ 3.35 mM) to 10.0 ± 0.6 cycles/min (K^+^ 8.00 mM); n = 10; p = 0.443; Fig. 5B, C).

**Fig. 6:**
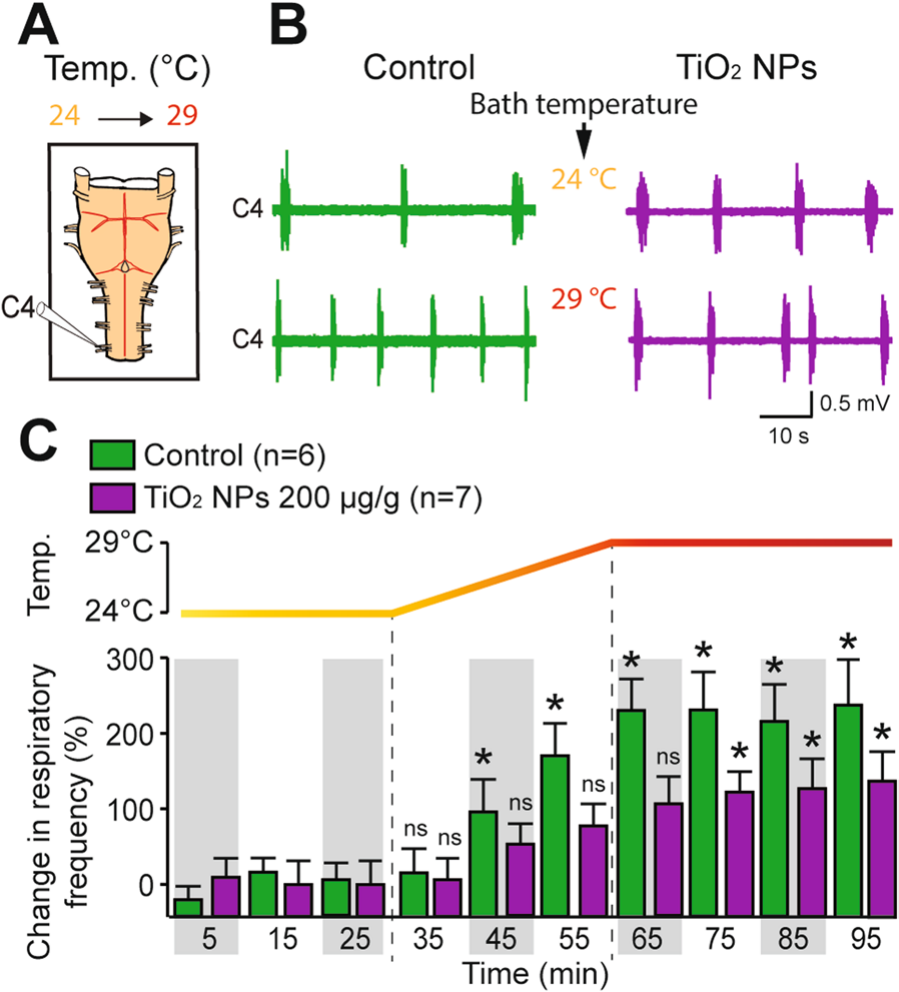
Effect of temperature on burst frequency of the respiratory CPG in unexposed and prenatally TiO_2_ NP-exposed neonates. **A** Schematic of experimental procedure. **B** Raw extracellular burst activity recorded from a C4 ventral root in isolated preparations from unexposed (Control, green traces) and prenatally TiO_2_ NP (200 µg/g)-exposed (purple traces) neonates under standard (24 °C, upper traces) and elevated (29 °C, lower traces) bath temperatures. **C** Bar charts showing changes in spontaneous respiratory burst frequency as a function of time (mean ± SEM) in control (green bars) and prenatally TiO_2_ NP-exposed (purple bars) groups under the warming protocol. Data were pooled from P0 to P3. *p < 0.05; ns, not statistically significant

These latter results are thus consistent with the idea that exposure to TiO_2_ NPs during gestation may indeed affect the capacity of the central respiratory network to respond adequately to excitability changes derived from the neonate’s external environment. To further explore this possibility, we performed a series of ex vivo experiments in which we assessed the ability of the respiratory CPG to produce an increased burst frequency in response to a more realistic physiological stimulus, namely that provided by a change in environmental temperature. Indeed, in addition to key mechanisms of thermolysis, such as skin vasodilatation and sweating, one crucial function of the respiratory system is to remove excess body heat by producing accelerating breathing when body temperature is augmented, as occurs during fever, exercise or high ambient temperature. In a first experimental approach, therefore, using isolated brainstem-spinal cord preparations, the bath temperature was progressively raised from 24 °C, the standard temperature in our ex vivo experiments, to 29 °C (Fig. 6A). Under this changing extrinsic condition, isolated CNS preparations from non-exposed control neonates (n = 6) showed a rapid, progressive and significant increase in respiratory burst frequency (Fig. 6B, C), which reached a steady-state plateau that corresponded to a twofold augmentation of the initial respiratory rhythm frequency as soon as the highest temperature was reached (Fig. 6C). This observation was in accordance with results previously found on a similar rat brainstem-spinal cord preparation [29–32]. In contrast, isolated preparations from prenatally TiO_2_ NP (200 µg/g)-exposed newborn mice failed to exhibit a significant respiratory burst frequency increase during the warming ramp, and only showed a delayed and moderate increased respiratory rate for the highest bath temperature (Fig. 6B, C). Consequently, the change in respiratory burst frequency in response to the 5 °C temperature increase was much less pronounced in preparations obtained from gestationally TiO_2_ NP exposed offspring compared to control (121 ± 17% vs 226 ± 24% rate increase, respectively; p < 0.001).

**Fig. 7:**
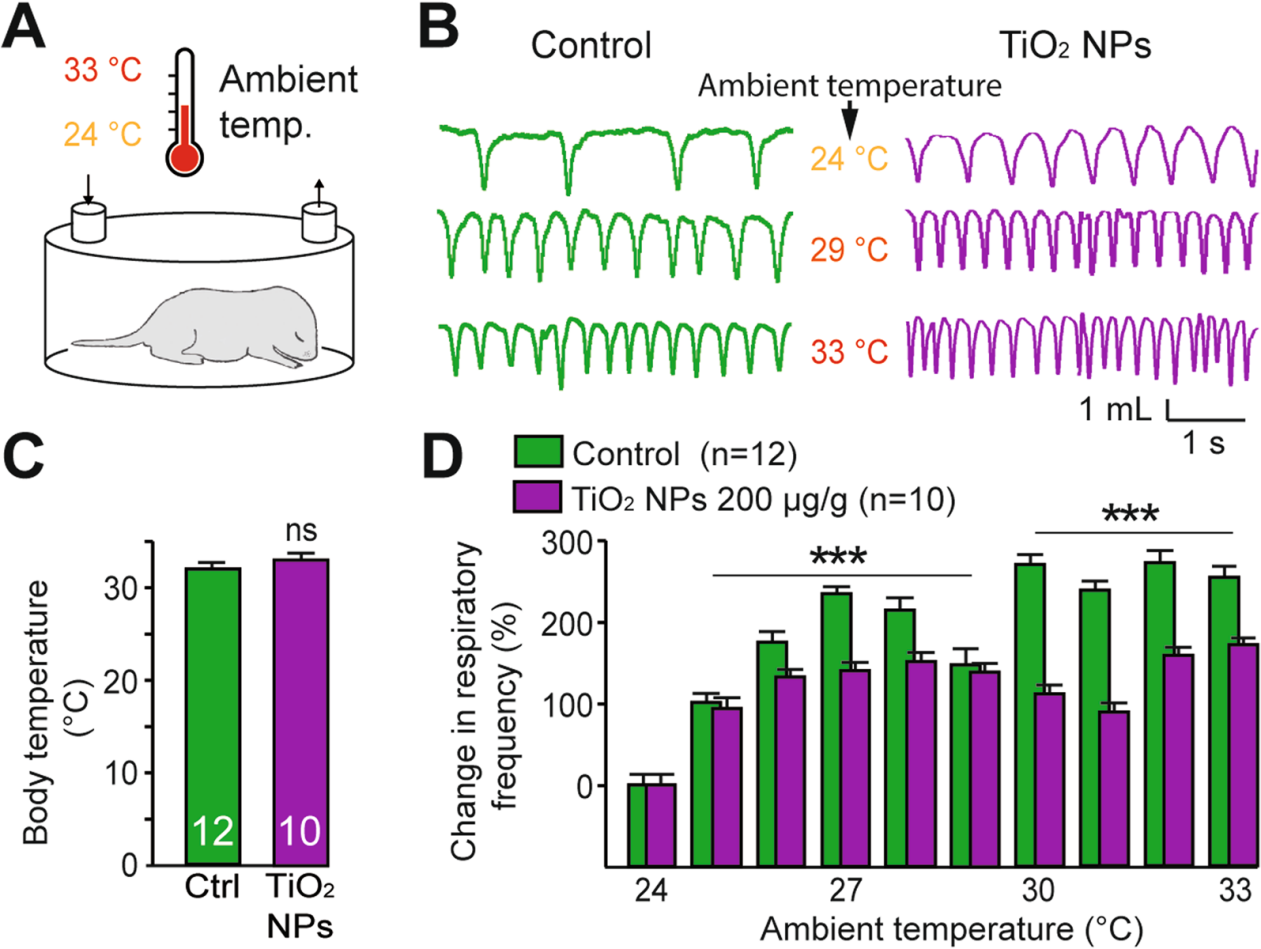
In vivo effect of temperature on breathing rate in unexposed and prenatally TiO_2_ NP-exposed neonates. **A** Schematic of experimental procedure. **B** Whole-body plethysmographic recordings of non-exposed (Control, green traces) and TiO_2_ NP (200 µg/g)-exposed offspring (purple traces) under different ambient temperature conditions (indicated between traces). **C** Bar charts showing values of body temperature under standard room temperature in control (green bar) and TiO_2_ NP (200 µg/g)-exposed offspring (purple bar). **D** Bar charts illustrating changes in spontaneous breathing frequency (mean ± SEM) in control (green bars) and prenatally TiO_2_ NP-exposed (purple bars) groups under the warming protocol. Data were pooled from P0 to P3. ***p < 0.001; ns, not statistically significant

In a final step, we asked whether these differences in the effects of temperature variations on respiratory CPG activity in reduced experimental preparations could also be detected in the actual breathing behaviour of intact animals. In vivo plethysmographic recordings from P0-P3 neonates (Fig. 7A) indeed revealed that prenatally TiO_2_ NP-exposed pups exhibited an inability to produce an appropriate breathing rate augmentation in response to an increase in ambient temperature. As already observed during exposure to 24 °C (control temperature), the breathing rate was significantly higher in prenatally exposed animals than in non-exposed controls (Figs. 2C, 7B). It is noteworthy that, at the initial ambient temperature (i.e. 24 °C), there were no significant differences in the body temperature measured from non-exposed control (n = 12) and TiO_2_ NP-exposed (n = 10) neonates (32.0 ± 0.7 °C *vs* 32.9 ± 0.5 °C, respectively; p = 0.359; Fig. 7C). When the ambient temperature was gradually raised from 24 to 29 °C, a significant increase in breathing frequency occurred in both groups, although less pronounced in TiO_2_ NP exposed offspring (non-exposed group, mean value 160 ± 7% increase; exposed group, 130 ± 5% increase; p < 0.001; Fig. 7D). However, prenatally TiO_2_ NP-exposed neonates were unable to further accelerate their breathing rate during a subsequent further rise in temperature to 33 °C (non-exposed group, 250 ± 6% increase; exposed group, 130 ± 4% increase; p < 0.001; Fig. 7D). Altogether, therefore, our ex vivo and in vivo results support the conclusion that a prenatal chronic exposure to TiO_2_ NPs impairs the capacity of the respiratory neural networks to satisfactorily adapt breathing rate to an excitatory environmental stimulus, such as a rise in temperature.

## Discussion

### Characteristics of TiO_2_ NPs

Our results show that the size of TiO_2_ NPs used in our experiments ranged from 5 to 50 nm, and that the anatase crystalline form was predominant (i.e. 80%). After inhalation or ingestion of NPs, it is widely accepted that the shape, size and crystalline phase of nanosized metal particles strongly influence their translocation in the body. Previous studies on rodents showed that after being intranasally instilled, TiO_2_ NPs translocated to various organs when particle size ranged from 30 to 100 nm [33]. In a recent distribution analysis, titanium content was shown to increase in the liver, brain and placenta after intra-esophageal instillation of TiO_2_ NPs [7]. Moreover, it has been reported that a significant fraction of anatase-type particles, known to have a higher capacity than the rutile polymorph for causing oxidative stress and cellular toxicity [34], passes the intestinal barrier only 1 h after oral administration. After fecal excretion, approximately 0.6% of a single administered dose crossed the gastrointestinal barrier, and about 0.05% of these particles remain distributed throughout the body 7 days after the initial exposure [35]. Although the systemic availability of TiO_2_ NPs is extremely low after oral administration, detectable NP concentrations are still observed in several organs including the lungs, uterus and brain [35, 36]. It has also been reported that a combination of anatase and rutile crystalline forms cause more severe cell damage than pure anatase or pure rutile TiO_2_ NPs [37].

Furthermore, our findings indicate that the surface charge of TiO_2_ NPs used in our experimental conditions (i.e. pH = 7.4) was negative. Whereas cationic NPs are more rapidly internalized in cells and more cytotoxic than anionic particles due to their attractive electrostatic interactions with the negative charge of the inner cell membrane [38, 39], it has been clearly demonstrated that anionic TiO_2_ NPs can influence the operation of membrane proteins such as voltage-gated ion channels in neurons [40], potentially facilitated by their attractive interactions with the positively charged outer cell membrane. Based on their physicochemical properties described above, therefore, the TiO_2_ NPs used in our study under conditions of repeated ingestion exposure, have the ability to cross biological barriers, to reach and accumulate in multiple organs, and to interact with different cellular targets with potential deleterious effects.

### Prenatal exposure to TiO_2_ NPs and breathing in newborn offspring

Maternal exposure to TiO_2_ NPs that can be shared with the fetus during development could potentially lead to a predisposition of newborns to develop long term environmental diseases from birth through adulthood. Previous studies in rodents have unequivocally demonstrated that a repeated exposure to TiO_2_ NPs through ingestion [7] or inhalation [41] during pregnancy increases titanium concentrations in the placenta, impairing the latter’s growth and development [6]. Either directly via a materno-fetal transfer of nanomaterials, or indirectly through placental or vascular dysfunction [42], the progeny’s respiratory system is also likely to be subsequently altered. Indeed, a recent study reported that maternal gestational exposure to TiO_2_ NPs induces a dysregulation of placental vascularization, inhibited the formation of fetal blood vessels [6] and impaired umbilical vascular reactivity [43]. Consequently, such impairments could lead to hypoxic conditions that force the fetal respiratory system, even after birth, into the expression of a reactionary, long-lasting increase in respiratory frequency associated with a decrease in the occurrence of apneas as observed in the present study. Another indirect mechanism to consider is that prenatal exposure to TiO_2_ NPs could trigger the production of biological mediators in pregnant mice, which in turn would induce an undesirable excitatory respiratory effect in the fetus. Indeed, recent studies clearly show that exposure to TiO_2_ NPs by oral intake [44–47], inhalation [48, 49] or dermal contact [50] generates pro-inflammatory responses and the production of various cytokines. Among them, some are known to activate the respiratory centers [51, 52] and could potentially act as a modulator of the respiratory system in the fetus. Furthermore, maternal exposure during gestation (by oral gavage) has already been found to produce long-lasting impairment of offspring lung development [13], including deficient septation, pneumocytic apoptosis, and macrophage infiltration [9]. Here, we report that a chronic maternal exposure to TiO_2_ NPs during the gestational period (by voluntary food intake) also impairs the normal development of the actual breathing rhythm in mouse offspring but without a noticeable structural alteration of their lung tissue (Additional file 2: Fig. S2). From birth until the end of the first postnatal week, which corresponds to the first two months of life in humans [53], breathing of prenatally-exposed neonates was characterized by an abnormally high respiratory rate associated with a near absence of apneas. Because maternal TiO_2_ NP treatment ended just prior to time of birth, we hypothesised that the disappearance of the TiO_2_-induced respiratory effect by P7 might be due either to a progressive decrease in tissue titanium levels, which is typically observed after exposure cessation [35, 54]. We also observed that this abnormal excitatory effect on postnatal breathing rate led in turn to a low variability in the interval between breath cycles. It is noteworthy that the rate variability of an autonomic rhythmic function can be considered as a predictor factor for dysfunction or pathology. For example, heart rate variability, that is the degree of fluctuation in the duration between two consecutive heart contractions, is commonly used as a mirror of the cardiac control system, and gives information about the risk for sudden cardiac death [55, 56]. Therefore, an increased breathing frequency coupled with a reduction in breath-to-breath interval variability was indicative of a rapid, highly stereotyped rhythmic activity that was likely to have a reduced capacity for respiratory adaptation, a process that is crucial for satisfying an organism’s changing oxygen demands or to satisfactorily adapt breathing rate to an excitatory environmental stimulus, such as an increase in body temperature. At birth, this lack of respiratory adjustment that we observed in our in vivo and ex vivo experiments could weaken a neonate’s chances of survival because of its increased vulnerability to environmental risk factors. Notably, it could facilitate the occurrence of excessive body heat gain in conditions of high external temperature, or participate in triggering an inappropriate response to hypoxia, hypercapnia or hyperthermia. These factors would in turn contribute to increase the risk of morbidity and mortality, especially in more fragile cases such as low birth-weight infants and preterm newborns [57–59].

### Potential neurotoxic effects of TiO_2_ NPs on offspring

Although indirect mechanisms as those mentioned above may be involved, our findings strongly suggest that in mice, a repeated maternal exposure to TiO_2_ NPs can directly alter the prenatal development of breathing by changing the excitability of the neural networks engaged respiratory rhythmogenesis. Indeed, several studies point toward a more direct action of NPs on offspring. In rodents [8, 60–62] and human [63], TiO_2_ NPs can cross placenta barrier (70 to 100% of TiO_2_ particles (< 200 nm) recovered in the fetal side [63]), accumulate in the fetus, that ultimately could result in direct neurotoxicity on offspring (for a review see reference [64]). In fact, an increasing number of studies have reported that neurotoxicity constitutes one of the major adverse effects of TiO_2_ NPs on rodent offspring. For example, inhalation exposure to TiO_2_ NPs can produce a dysregulation of blood brain barrier physiology associated with neuroinflammation [65]. Furthermore, maternal exposure to these NPs, either by repeated subcutaneous injections [66] or by inhalation during gestation [67] can affect the expression of certain genes involved in the development and operation of the central nervous system, causing in neonatal mice alterations in the cerebral cortex, olfactory bulb and certain regions associated with the dopaminergic system [14, 15]. A significant decrease in brain-derived neurotrophic factor, crucial for growth and differentiation of neurons and synapses, and known to play an acute role in the development and modulation of respiratory rhythmogenesis [68–70], has also been reported in neonates born from rats orally-exposed to TiO_2_ NPs [71]. Additionally, it has been shown that such a maternal exposure by ingestion causes a decrease in cell proliferation [62] and retardation of axonal and dendritic outgrowth in offspring [72], and in the most critical cases, early neuronal death affecting in particular the hippocampus [73]. In light of these experimental findings, the question then arises as to what the consequences might be on a short- (childhood), middle- (adolescence) and long-term (adult) scale for brain function. From birth, in relation to alterations observed in the hippocampus, mouse neonates exposed to TiO_2_ NPs during the fetal period (via maternal ingestion exposure) show a significant degradation in learning and memory performance [10]. Without apparently affecting the normal growth of progeny, maternal exposure to TiO_2_ NPs by a single intravenous injection during gestation can lead to subtle and dose-dependent neurodevelopmental disorders as revealed, for example, by a significant decrease in neonatal mouse vocal communication and juvenile sociability, two markers of autism spectrum disorders [74]. Finally, regardless of the maternal exposure route to TiO_2_ NPs during gestation, impairment of cerebral function is also observed in prenatally exposed mice after reaching adulthood. These findings thus reinforce the idea that very early exposure during development favours the occurrence of certain deficits or pathologies in adulthood such as an impairment of learning and memory after maternal ingestion [62], deficiency in motivation [75] and a tendency to avoid possible stressful situations following maternal inhalation exposure [76], or depressive-like behaviors after maternal subcutaneous injection [16].

Besides such behavioural and cognitive dysfunctions, our study reports an adverse effect of TiO_2_ NPs on the neural networks involved in a vital motor function. The precise mechanism by which TiO_2_ NPs induce elevated respiratory-related burst frequency in prenatally exposed offspring is still unknown and now requires further investigation. Although an indirect action via an abnormal activation of excitatory neuromodulatory systems cannot be excluded, since such systems can be disrupted in the fetal brain by a prenatal exposure to TiO_2_ NPs [14], a direct action on neurons belonging to the pre-Bötzinger complex, the network center controlling respiratory rhythmogenesis [26, 27], is also a possibility. Indeed, our results clearly show that when isolated in the brainstem-spinal cord preparation, and even more drastically in medullary slices obtained from prenatally-exposed neonates, the pre-Bötzinger network generates an abnormally elevated respiratory-related rhythm that resembles the increase in breathing rate observed in the intact animal. Intriguingly, spontaneous respiratory burst frequency in brainstem-spinal cord preparations from neonates exposed to the two lower doses (50 and 100 µg/g) were not significantly different from control (see Fig. 4C), despite their significant accelerating effect on breathing previously observed in vivo (see Fig. 2D). A plausible explanation for this discrepancy, however, is that exposure to low doses of TiO_2_ NPs affects respiratory function mainly through a peripheral action on neural processes (sensory inputs, for example) that are absent from our ex vivo preparations. On the other hand, high doses could produce their adverse respiratory effects through a combination of both peripheral actions and central effects on the respiratory CPG circuitry itself. Therefore, and without excluding other possible contributing factors, these correlated findings strongly suggest that exposure to TiO_2_ NPs induces a long-lasting modification in the excitability of respiratory CPG network neurons, possibly via changes in their intrinsic bioelectrical properties.

## Conclusions

This study provides insights into the deleterious effects on offspring of a chronic maternal exposure to TiO_2_ NPs on the development of breathing, a vital motor function. We demonstrate that exposure to TiO_2_ NPs during the perinatal period somehow affects bursting activity within the central neural networks involved in respiratory rhythm generation. In their fields of application, NPs used in this study (P25-type) have been developed mainly for technical use such as in photocatalysis, metal coating or semiconductor membranes. Although they are also present in cosmetic products (sunscreens, lipsticks, toothpaste, etc.) that may inadvertently enter the gastrointestinal tract, P25 NPs are generally considered to be little ingested, in contrast to TiO_2_ E171, which is a common additive in food products. Because reference TiO_2_ P25 and food-grade TiO_2_ E171 have been shown to behave differently in their environmental interactions [77], and thus potentially have different toxicities [78], further studies will be needed to assess the potential deleterious effect of E171 on the development of breathing in the offspring. Nonetheless, using brainstem-spinal cord preparations and medullary slices from neonatal rat, we have also previously shown that an acute exposure to zinc oxide NPs accelerates, then severely depresses burst activity generated by the pre-Bötzinger complex responsible for inspiratory rhythmogenesis [79]. The present study on chronic gestational exposure to TiO_2_ NPs thus reinforces our working hypothesis concerning the toxic effect of certain metal oxide NPs on the developing respiratory neural networks. In the framework of the exposome concept [1], moreover, our present report complements studies carried out in children where the toxic effects of pre- and postnatal exposures to pollutants on lung development and breathing troubles in newborns have been evaluated [80, 81]. Although our present conclusions cannot be immediately extrapolated to humans, they do raise concerns about the fetotoxicity of TiO_2_ NPs in light of their growing use in everyday consumer products.

## Methods

### Newborn animals and care

Experiments were conducted on pregnant and neonatal CD1 mice. Newborn animals (0 to 7 day-old, of either sex) were obtained from females raised in our laboratory’s breeding facility. Pregnant mice (7–8 week-old) were kept in a humidity-controlled room on a 12 h/12 h light–dark cycle. Standard basal food and water were provided ad libitum.

### Titanium dioxide nanoparticles (TiO_2_ NPs) and transmission electron microscopy (TEM)

Our experiments were conducted using TiO_2_ P25 NPs obtained from Sigma Aldrich (ref. 718,467). The structure (shape and size) of TiO_2_ NPs was examined by TEM first by suspending particles in ethanol, then transferring them in a drop of this solution to a 200 mesh formvar coated grid, and left to dry. Grids were observed with a Hitachi H7560 TEM at 100 kV. TEM images were obtained with a TEM 1400 JEOL using 120 kV voltage operating at 120 kV. To be representative the statistics were performed on at least 250 NPs.

### X-ray diffraction and evaluation of Zeta potential

The phase identification of samples was performed with X-Ray Diffraction using a powder diffractometer (PANalytical X’Pert Pro) equipped with CuKα1 radiation (λ = 1.540598 Å). The Zeta potential evaluation was made using Nanoplus Micromeritics. Two stock solutions were prepared, one for measurement in basic pH and the other in acidic pH. The pH was adjusted with 0.001 mol L^−1^ and 0.01 mol L^−1^ HNO_3_ and NaOH solutions, respectively, in order to maintain the NP dilutions identical for each measurement. Samples were analysed between pH 2 and 10.

### Maternal exposure to NPs

Exposure to TiO_2_ NPs was performed daily throughout pregnancy. From the first gestational day (determined by the presence of a vaginal plug the morning following the mating night) until delivery, TiO_2_ NPs contained in chocolate spread were added to food and administered by voluntary food intake. This exposure protocol reduced, or even eliminated, any stressful situation for the animals, and guaranteed very precise control of the doses ingested by the pregnant mice. Then, pregnant mice were divided into a non-exposed control group (receiving chocolate spread only), and TiO_2_ NP (50, 100 and 200 µg/g body weight)-exposed groups (see next paragraph). Pregnant mice were weighed daily, as were newborn pups who also had their body length measured daily from birth until the end of the first postnatal week.

It is noteworthy that the dietary exposure of humans to TiO_2_ has been estimated to be up to 1.1 and 2.2 mg/kg body weight/day in the United Kingdom and United States, respectively [4]. Moreover, in a recent reappraisal, the European Food Safety Authority estimated that an average adult is typically exposed to 0.3–3.8 mg TiO_2_/kg body weight per day [82]. Although the highest daily dose used in our study was approximatively 50-fold greater than that estimated in humans, the total amount of TiO_2_ NPs delivered to a pregnant mouse over the ~ 19 day gestation was effectively 500-fold less than the estimated quantity taken in by a woman during the entire duration of her pregnancy (~ 280 days).

### Whole body plethysmographic recordings

The breathing rate was measured from unrestrained neonates using a whole-body plethysmograph. Plethysmographic recordings were performed at the Animotion collaborative core facility of the INCIA laboratory (CNRS UMR5287, University of Bordeaux, France). P0–P7 newborn mice were placed inside a 50 ml chamber for recording sessions each lasting 5 min. The chamber was placed under a heating lamp and maintained at a constant temperature of 24 °C throughout the entire recording period. In the chamber interior, the air was continuously renewed by a pump with a constant flow (0.8 L/min). The chamber was connected to a data acquisition system (Emka Technologies) that measured flow and pressure changes within the chamber using Iox2 software. Data were sampled at 1 kHz and recordings were analysed offline using Spike2 software (Cambridge Electronic Design). Breathing rate was calculated on a breath-to-breath basis. By convention, each inspiratory phase was indicated by a negative volume, expiratory phase by positive one. Interruptions in the breathing rhythm were considered as apneas when at least 3 ongoing cycles were skipped.

In some experiments, the breathing rate of neonates was measured as a function of the ambient temperature that was progressively raised from 24 to 33 °C using a lamp heat source. To this end, the lamp was gradually brought closer to the animal and a temperature probe positioned in the chamber was used.

### Ex vivo preparations and electrophysiological recordings

The experimental procedures have been previously described in detail [79, 83, 84]. Ex vivo experiments were first performed on isolated preparations of the brainstem and spinal cord of 0- to 3-day old mice. Neonatal animals were anesthetized with 4% isoflurane for 10 min until the loss of reflex responsiveness to tail pinching. Animals were then decapitated and eviscerated. The skin and muscles were removed and preparations were placed in a 25 ml chamber containing circulating (flow rate, 5–10 ml/min) artificial cerebrospinal fluid (in mM: 125 NaCl, 3.35 KCl, 0.58 Na_2_HPO_4_, 1.26 CaCl_2_, 1.15 MgCl_2_, 21 NaHCO_3_, 30 D-Glucose; equilibrated with 95% O_2_/5% CO_2_, pH 7.4). The entire brainstem and spinal cord were carefully isolated with its ventral roots still attached. The temperature of the saline was set at 24 °C before recording procedures began. For some experiments, spontaneous respiratory-related activity was measured during a progressive increase in bath temperature up to 29 °C (temperature ramp rate 1 °C/6 min) by means of a Peltier device. In some other experiments, the spinal cord was exposed to a modified saline solution containing a high potassium concentration (8.00 mM KCl) to reversibly increase neural excitability.

To obtain medullary slices containing the pre-Bötzinger complex (preBötC), the rhombencephalon of neonatal mice was isolated by sectioning its rostral limit and the spinal cord at the upper cervical level. The preparation was then embedded in an agar block, mounted on a vibratome and serially sliced in the transverse plane in a rostral-to-caudal direction until the posterior limit of the facial nucleus and the anterior limit of the nucleus ambiguus were reached. A 550 µm-thick slice was transferred to a recording chamber, continuously superfused with aCSF maintained at 30 °C and containing (in mM): 120 NaCl, 8 KCl, 1.26 CaCl_2_, 1.5 MgCl_2_, 21 NaHCO_3_, 0.58 NaH2PO_4_, 30 D-glucose, buffered to pH 7.4 with NaOH and saturated with 95% O_2_ and 5% CO_2_.

Respiratory-related burst activity occurring in ventral roots or directly in the pre-Bötzinger networks was recorded using glass suction electrodes filled with artificial cerebrospinal fluid solution. Signals were amplified (× 10,000) by differential AC amplifiers (low cutoff, 100 Hz; high cutoff, 1 kHz, model 1700; A-M Systems), digitized and acquired via a CED 1401 plus interface, stored on a computer, and analysed using Spike2 software (Cambridge Electronic Design).

### Lung staining

Lungs were removed and fixed in 4% PFA overnight, then in a 20% PBS-sucrose solution. They were subsequently frozen at − 80 °C and 40 µm thick sections were prepared with a cryostat and carefully recovered and positioned on slides. Then, the slides were immersed in PFA 4% overnight, and transferred through a graded ethanol series (50°, 70°, 95° and 100°) to dehydrate and rehydrate the sections. Cresyl violet stain was deposited on the slides for 5 min then the latter were re-immersed in the alcohol baths to dehydrate tissue. To fix the stain and clear tissue, the sections were then immersed in a xylene bath. Finally, slides were mounted with mounting medium and observed under a microscope (Leica fluorescence microscope).

### Statistical analysis

Statistical analyses were carried out with SigmaPlot 11.0 (Systat), with values being expressed as mean ± SEM. A Student’s t test or Mann–Whitney test for non-normal distribution was used to compare the means of two groups. To compare more than two groups, an ANOVA followed by a Turkey’s post-hoc test, or an ANOVA on ranks (i.e., Kurskal Wallis test) followed by Dunn’s post-hoc test were used. Differences were considered statistically significant when the p value was < 0.05.

## Supplementary Information

**Additional file 1: Fig. S1**. Weight and size of pregnant mice and offspring in non-exposed and prenatally TiO_2_ NP-exposed groups. **A–C** Bar charts (mean ± SEM) showing weight gain of pregnant mice (**A**), number of offspring per litter (**B**) and weight gain per neonate (**C**) in non-exposed control (green bars) and prenatally TiO_2_ NP-exposed (purple bars) groups. The number of animals is indicated in each bar. **D**, **E** Scatter plots illustrating changes in weight (**D**) and size (**E**) of the neonates during the first postnatal week under these four experimental conditions. Shaded areas in D and E, which are delimited by ± 2 SEM of the mean of non-exposed animals, represent normal postnatal growth. The mouse image is from Servier Medical Art website (smart.servier.com). ns, not statistically significant.

**Additional** **file** **2:** **Fig. S2**. Histological and morphological study of the lungs of non-exposed and prenatally TiO_2_ NP-exposed neonatal mice. **A** Photomicrographs of stained (cresyl violet) lung sections from non-exposed (top) and TiO_2_ NP (200 µg/g)-exposed (bottom) neonates. Sections (40 µm thick) were made with cryostat. **B–D** Bar charts showing quantification of lung/body weight ratio (**B**), alveolar density (**C**) and lung size (**D**) in control (Ctrl, green bars) and prenatally TiO_2_ NP-exposed (purple bars) neonates. The number of animals is indicated in each bar. ns, not statistically significant.

## Data Availability

The datasets used and/or analysed during the current study are available from the corresponding author on reasonable request. All data generated or analysed during this study are included in this published article and its supplementary information files.

## Abbreviations

CNS: Central nervous system
CPG: Central pattern generator
NP: Nanoparticle
preBötC: Pre-Bötzinger complex
TEM: Transmission electron microscopy
TiO_2_: Titanium dioxide
