# Supplementary figures and images for: Chronic maternal exposure to titanium dioxide nanoparticles alters breathing in newborn offspring
Source: Part Fibre Toxicol. 2022 Aug 18;19:57. doi: 10.1186/s12989-022-00497-4 (PMC9386967; doi:10.1186/s12989-022-00497-4)

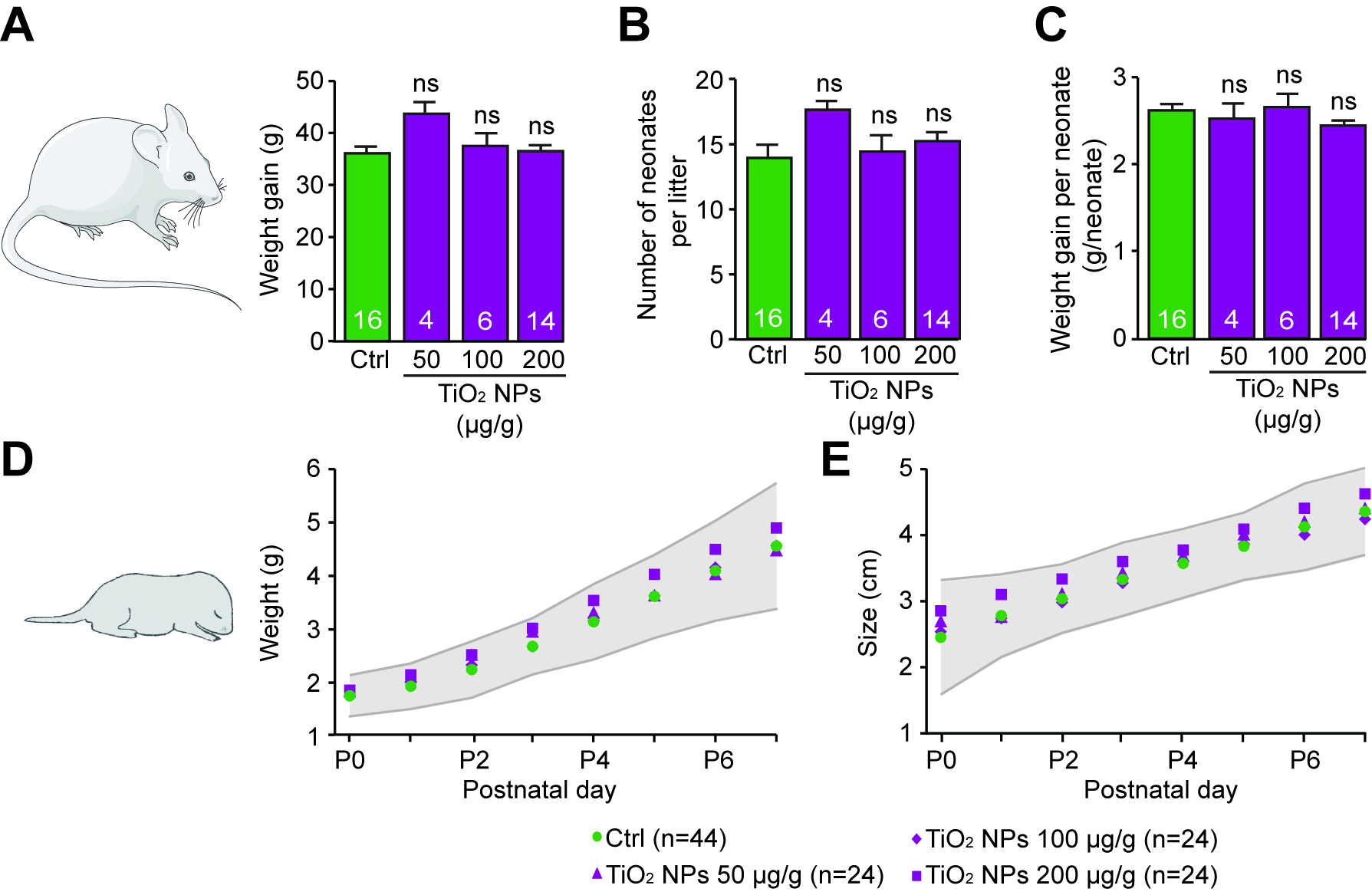

Supplement: Supplementary file 1 — Additional file 1: Fig. S1. Weight and size of pregnant mice and offspring in non-exposed and prenatally TiO2 NP-exposed groups. A–C Bar charts (mean ± SEM) showing weight gain of pregnant mice (A), number of offspring per litter (B) and weight gain per neonate (C) in non-exposed control (green bars) and prenatally TiO2 NP-exposed (purple bars) groups. The number of animals is indicated in each bar. D, E Scatter plots illustrating changes in weight (D) and size (E) of the neonates during the first postnatal week under these four experimental conditions. Shaded areas in D and E, which are delimited by ± 2 SEM of the mean of non-exposed animals, represent normal postnatal growth. The mouse image is from Servier Medical Art website (smart.servier.com). ns, not statistically significant. [file 12989_2022_497_MOESM1_ESM.tif]

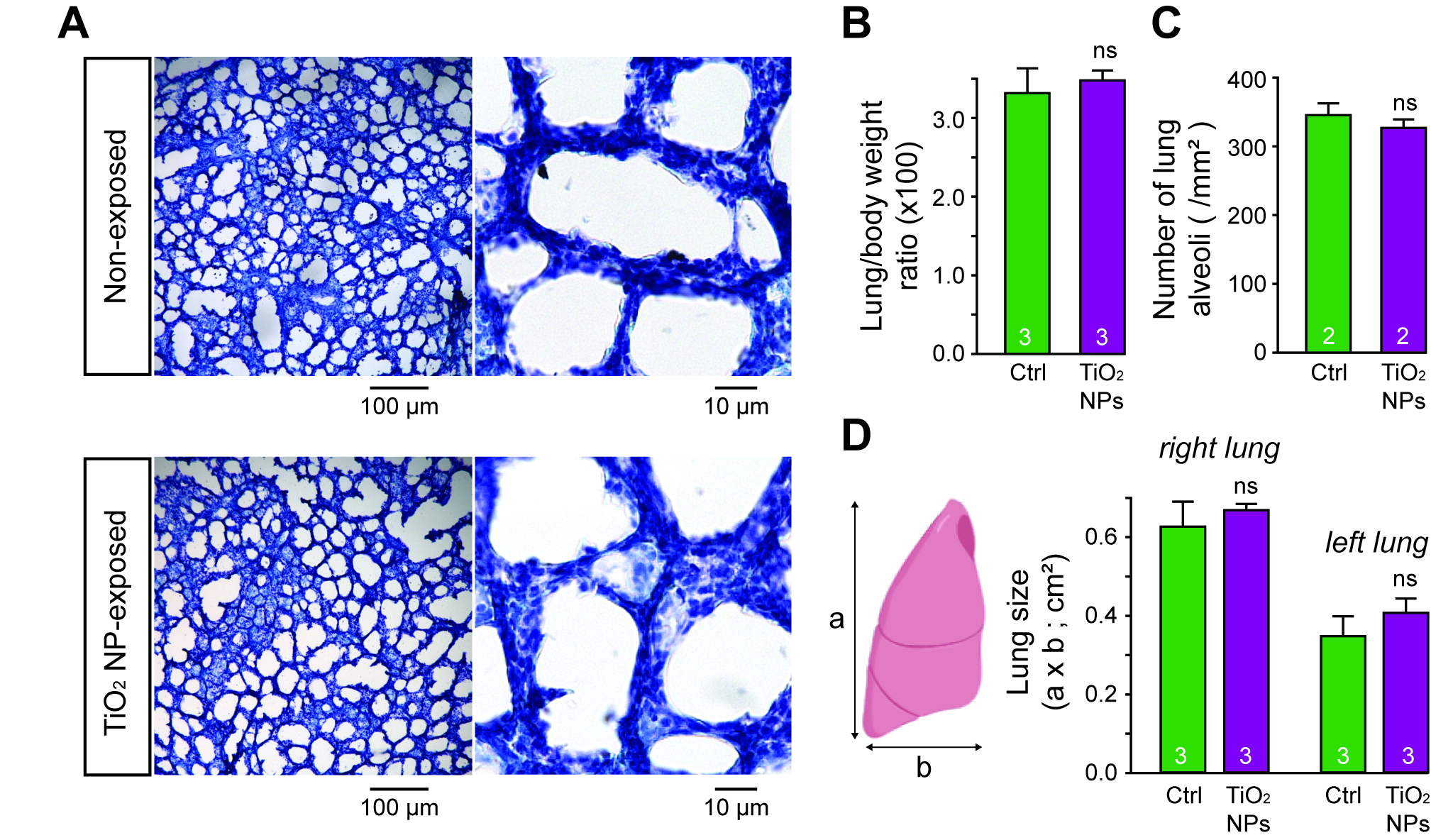

Supplement: Supplementary file 2 — Additional file 2: Fig. S2. Histological and morphological study of the lungs of non-exposed and prenatally TiO2 NP-exposed neonatal mice. A Photomicrographs of stained (cresyl violet) lung sections from non-exposed (top) and TiO2 NP (200 µg/g)-exposed (bottom) neonates. Sections (40 µm thick) were made with cryostat. B–D Bar charts showing quantification of lung/body weight ratio (B), alveolar density (C) and lung size (D) in control (Ctrl, green bars) and prenatally TiO2 NP-exposed (purple bars) neonates. The number of animals is indicated in each bar. ns, not statistically significant. [file 12989_2022_497_MOESM2_ESM.tif]
